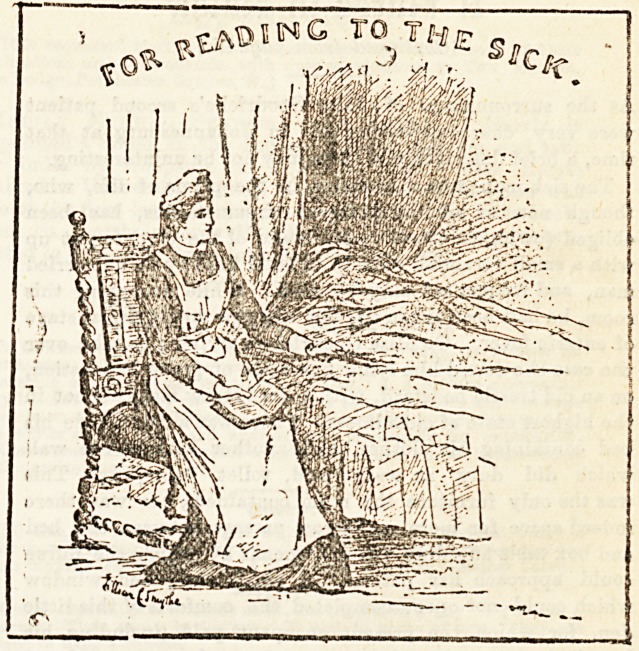# The Hospital Nursing Supplement

**Published:** 1891-12-05

**Authors:** 


### "The Hospital" Nursing Mirror.

Being the Extra Nursing Supplement of "The Hospital" Newspaper.

Contributions for this Supplement should be addressed to the Editor, The Hospital, 140, Strand, London, W.C., and should have the word "Nursing" plainly written in left-hand top corner of the envelope.

### En Passant.

THE NURSE-DOLLS. — Miss Pigott, of the Royal Infirmary, Preston, has asked the loan of the Nurse-Dolls for her big doll show which is to come off in December, and we have gladly sent a selection of the dolls, for the cause is such a good one. We had not intended to let the dolls travel any more, they looked so tired and soiled after their journeyings, but unfortunately their case is not air-tight, and London smoke was disagreeing with them, so we were glad to send them for a temporary change, hoping to manage a better arrangement for them on their return. The dolls have another invitation to go to Burslem for January, which we hope we shall be able to accept for them. What a good work the dressers of these dolls did! They have helped so many charities.

THE LATE LORD LYTTON AND HIS NURSE.—It is a comfort to think that the late Lord Lytton was tended in his last illness, and on several previous occasions, by English trained nurses, albeit in a foreign city. Her Ladyship always took the greatest interest in the Levick Nursing Institution in Paris, as conducing to the comfort and well-being of English-speaking travellers. And her kindly intention was the means of procuring skilled attendance around his Lordship's sick bed. Almost the last words which Lord Lytton spoke were "Good night, dear Nurse, God bless you." This nurse, who was a great favourite with him, was called up immediately when he was taken suddenly worse, and arrived in time to smooth his dying pillow. Lady Lytton has presented Nurse Curphy, the nurse alluded to, with a copy of Lord Lytton's poems, and we sincerely congratulate her on this well-deserved recognition.

NURSING IN INDIA.—Since the Queen conferred the Royal Red Cross on some of our Sisters in India, we have been inundated by letters about the Indian Nursing Service. All correspondents will please find their queries answered here. The Indian Nursing Service is a branch of the Army Medical Department, and it is not necessary for a Sister to have served at Netley first. There is no "training" at Netley as one correspondent suggests; candidates have to train for three years in some large hospital, and then apply to the Director General, Army Medical Department, Victoria Street, S.W. They have to be of good social position. There are no such things as "Royal Red Cross Nurses," the nurses serving in the Army and Navy are known as Her Majesty's Nursing Sisters. We refer our correspondents to "Our Indian Letter," in our issue for September 19th; "Another Letter on India," in our issue for October 3rd; and "Our Indian Letter," on November 21st. For particulars about Lady Roberts' Fund see our issue for February 7th. During this year we have given eleven articles or notes on nursing in India, and we really cannot keep on repeating the same information week after week; our correspondents must take the trouble to look up back numbers. Nor can we answer queries on general subjects privately, our letters are terribly numerous as it is. Full information on this and all branches of nursing can be found in Burdett's "Hospital Annual."

ST. JOHN'S HOME.—The pupils who were sent up for the London Obstetrical Society's examination from St. John's Maternity Home, Battersea, have gained the diploma of that Society: Miss Kennedy, Mrs. Sibbery, Miss Washington, Miss Aimée Wieté, Miss Wiffin, and Miss Wills. This completes the roll for the year, during which time twenty-one candidates have been sent up for examination, all of whom have passed.

SHORT ITEMS.—One of the most beautifully kept graves in the Grange Cemetery at Edinburgh is that of Nurse Josephine Wade; it is always decked with flowers, and on the pretty marble cross is inscribed, "Until the day break, and the shadows flee away."—A doll show will shortly be held in aid of the Middlesboro' Nursing Association.—A new asylum, to hold 1,000 patients, is going to be built by the Metropolitan Asylums Board.—Mrs. St. Horton reports 128 admissions to her Home at Worthing from May to October.—Since the Princess of Wales has returned and taken a share in the nursing of her son, the health of Prince George has steadily improved.—Last week's *Queen* had a portrait of Miss Hallam, who trained at St. Mary's, and is in attendance on Prince George; Miss E. K. Ward, the other nurse at Marlborough House, has worked at Nottingham, St. Bartholomew's, and Nice. Both owe their present post of trust to Dr. Broadbent.—Miss Hewlett, Superintendent of St. Catherine's Hospital at Amritzer, India, is now in England lecturing in aid of that institution.—Miss Moon is delivering lectures on Hygiene to the members of the Liverpool Ladies' Sanitary Association. — At Edinburgh, when a sermon was being preached last Sunday in aid of the Royal Infirmary, a nurse got up and said she had served the Infirmary for fifteen years, and was now asked to resign, and she demanded an inquiry into the matter.

ST. MARY'S HOME.—For some reason (we hope merely from an oversight) no notice of the opening of St. Mary's Home at Plaistow was sent to us, and we, therefore, quote the following from the *Daily News*: "Lady Maud Wolmer, in the unavoidable absence of the Duchess of Westminster visited Plaistow yesterday afternoon, and opened the St. Mary's Nurses' Home and Cottage Hospital. The institutions are included in a series of six in connection with the Church and Parish of St. Mary's, Plaistow, which have been erected within the last three years for the benefit of the poor of a parish which, with more than 21,000 inhabitants, is one of the poorest near London. The Rev. Given-Wilson gave an account of the work done in the parish. He regretted the absence of the Duchess of Westminster, who is the Patroness of the Nursing Institution, and gave a cordial welcome to Lady Maud Wolmer, who then declared the home open. A procession, headed by a number of local clergy and the choristers of St. Mary's, in their surplices, was then formed, and proceeded a distance of about fifty yards to the Cottage Hospital. Lady Maud, having inspected the various wards, which presented an appearance of scrupulous cleanliness and perfect order, also declared this institution open. The procession then again formed and continued its route to an enormous tent adjoining the hospital where a sale of work in aid of the funds of the hospital was opened."

### The Pen-Bron Hospital.

*Translated by permission from the French of M. Pierre Loti.*

I find myself pleading for this cause with a kind of astonishment. The subject seems so foreign to me, and, at first, I confess it almost repelled me. What surprises me most is the earnestness with which I write, my genuine desire that others should be informed, convinced, and filled with enthusiasm as I was. This autumn I received a letter from a certain much-honoured Admiral, in which he asked me to interest myself in the Pen-Bron Hospitals. I had never before seen the name, and I confess that, but for the signature at the end of the letter, I should have thrown it aside. What did they want of me, and what was their object? A Hospital for Scrofulous Children! How could that concern me, of all men? Better far leave the poor little creatures to die, and save them thus from a life of misery, and from producing, maybe, an enfeebled offspring. There are, alas! enough consumptives in France, enough weaklings in our army!

However, out of respect for my correspondent, I replied that I would make an effort, and do my best. Then I wrote, rather reluctantly, to M. Pallu, the founder of Pen-Bron—whose name and address the Admiral had given me, and said that he could dispose of my services.

Two or three days later M. Pallu arrived from Nantes to call upon me. His eager words did not at once move me; the sickly scrofulous children of whom he spoke, still only filled me with vague horror, only awoke in me a sort of relative pity mixed with unconquerable disgust. I listened resignedly. He told me that some of his patients, their flesh eaten away by dreadful wounds, were brought to him with limbs enclosed in splints; that others were carried in little boxes, and were literally almost falling to pieces; that after a few months he was able to set them on their feet, to remake their bones, to give them back a kind of health, and ensure prolonged existence. At this point I grew weary, I interrupted him to say, "It would, perhaps, be more humane to let them die."

Very calmly he replied that there he agreed with me. Then I began to foresee that we might come to an understanding. Possibly there was a deeper significance in his work than he had hitherto shown me; wider horizons which as yet I hardly divined. Little by little he told me strange and terrible things concerning the growth of this malady, called by a name which in itself is a stigma, of its increasingly rapid progress, especially within the last few years, of the sufferings and consequent physical deterioration of the children in our great cities, and added that a third of the population of France was tainted with the evil.

The cures effected at Pen-Bron on children hitherto pronounced hopeless, and certain to remain through life deplorably feeble, had for him no scientific value but as tests. They proved to him beyond a doubt, that this evil, I will not again name it, was absolutely, undeniably curable by salt air and sea in certain conditions of climate. It was his dream to extend the work indefinitely, so that it should assume immense proportions and rehabilitate the entire race.

"In this hospital," said M. Pallu, "which barely suffices for a hundred children, and which was founded by us with the greatest difficulty, we receive the dregs principally of other French hospitals. The cases are phenomenally diseased; almost invariably these unhappy children have lain for years in bed, have tired out every doctor, and have been brought to us *in extremis* when hope has departed. But would that, instead of a hundred children, we could receive thousands in great buildings, planned by the kilometre, with façades stretching their length across that marvellous sandy peninsular, with its mild climate and its salt air. Would that, instead of the little creatures we now have whose flesh is honeycombed by disease, they would bring us others in the earliest stage of the evil or whom it only threatens! If only each year all those pale and unhealthy children who languish in the close air of factories in our great cities, who later, scarred by disease, become but feeble soldiers, and whose sons will be more pitiable still; if all these, at an age when the constitution has such recuperative power, would turn to the sea and borrow from it a little of the strength it gives to sailors and fishermen!" Gradually, as he developed his idea, unfolding his thought with the ardour of conviction, I saw his eyes glow with an apostolic light, and I felt that the work to which he had consecrated his life was a noble one, truly patriotic and humane. Almost won over to his cause, I therefore promised, for I have never been able to write about what I have not thoroughly seen, to go to Pen-Bron before trying to write about it, and to see for myself, on those "marvellous sands," as he called them, what he had attempted to do.

. . . . . . . . . . . .

Some weeks later, towards the close of September, we were together at Le Croisie. The harbour was crowded with fishing-boats, and the sea was of that intense blue only observable in places where, through the presence of certain currents, it is more than usually salt and warm. Far away beyond the first blue bands were some sands which seemed to form an island, and upon them stood, in complete isolation, an old turreted châlet with freshly whitened walls. This châlet is Pen-Bron, but never did hospital look less like one. Indeed, one finds it hard to realise that that gay abode, open to all the winds, can enclose so many poor hapless ones, such excessive and rare forms of a horrible infirmity.

A boat took us across in a few minutes, and set us down on the sands, which are no islet, as would seem from a distance, but the extreme end of a very long and narrow peninsular, of a kind of limitless beach, wedged in between the ocean and some salt lagoons supplied by the sea.

Pen-Bron is there, surrounded by water like a ship. Beyond its walls, a garden has been extemporised, and though swept by blasts from the open sea, flowers grow all the same in its sandy borders. About sixty children are out of doors, little boys and girls in two separate groups.

The little boys sing, talk, and play together. The little girls are doing the same, watched by a good Sister in a mob cap, with the exception of a few rather older ones, who, seated on chairs, are working with the needle. And that, it seems, unless it rains heavily, is what they do every day. The boarders of Pen-Bron camp out of doors, constantly revolve round the walls of their house according to the way of the sun and the wind, sometimes facing the lagoon, sometimes the open sea, but inhale always that breeze which leaves a taste of salt on the lips. And, indeed, one might imagine himself in some boarding school at the recreation hour, but for a few crutches made to support weak little legs, a few bandages arranged so as to hide half the face, and three or four little armchairs set against the wall, which are rather painfully suggestive in shape. What I see is so reassuring that suddenly the kind of physical shrinking of unreasoning distress which seemed to contract my heart as I drew near this museum of horrors, takes to itself wings and disappears. Now as I approach the little patients I have only a feeling of interested curiosity; in the distance I can see them at play, like any other children of their age.

If, however, each of them were not tainted to the very marrow of his bones with some frightful disease he would not be here.

(*To be continued.*)

### The Victorian Exhibition.

THERE is now open at the New Gallery, Regent Street, an exhibition of articles of interest connected with the first fifty years of Her Majesty's reign. There is a fine collection of portraits including those of Mrs. Fry, Mrs. Browning, George Eliot, and other noted women. There is a case of surgical instruments as used in 1837, and another as used in 1887; there is a collection of medals, of postage stamps, of literary and artistic bric-a-brac, and of specimens of photography. There are most interesting relics of General Gordon, Thackeray, and Dickens; and of the progress in science as illustrated by our railways, telegraphs, and telephones. Nurses will find the exhibition both entertaining and instructive, though they will look in vain for the contrasted photographs of the nurse of fifty years ago and the nurse of to-day.

### The Screen for the Princess of Wales.

NEARLY 200 nurses visited St. Thomas's on Monday last, to view the screen which the Pension Fund members are presenting to the Princess of Wales as a birthday gift. The screen is of white wood, and of elegant design, and encloses 642 photographs. The effect is very pretty and light, and the workmanship is excellent. Together with the screen goes a book bound in vellum, and arranged in panels similar to those of the screen, only the names are in the book in the places occupied by the photos. There is also an illuminated address. A drawing by Mr. Reginald Cleaver of the nurses at Marlborough House occupies the centre of the middle panel, and round it are photographs of the four lady Patronesses of the Fund: Lady Rothschild, Lady Strafford, Lady Cadogan, and Lady Beaufort. Everyone who saw the screen expressed their admiration, and seeing that it is a humble tribute of admiration, and a bearer of good wishes from a band of working women to their Royal President, we are sure the Princess cannot but be pleased to receive it. The sympathy and thoughts of all nurses have specially been with Her Royal Highness during these last days, while she has herself been engaged in tending the sick in the person of her own son. May the nursing of the President of the Royal National Pension Fund rapidly restore to health our Sailor Prince.

### The Survival of the Fittest.

CYRIL BENNETT writes: The destruction of the physically weak has long been rigorously carried out by the natives of certain islands in the Pacific. The march of civilization having brought firearms within reach of these barbarous people, and the moral feelings which would have protected them being utterly undeveloped, the various tribes are busily exterminating one another like Kilkenny cats. *Vide* "A Naturalist among the Head Hunters," C. M. Woodford.

### A Bed for a Sick Nurse.

THE following sums have been received for the bed at the Brassey Home: Doris, 2s. 6d.; Sympathy, Harlow, 2s. 6d.; A Nurse's Friend, 2s.; Sister A., 2s. 6d.; Rickmansworth, 5s.; and A Patient, per No. 1,921, 5s. This money will be used for an easy chair, or some pretty article of furniture for the room, unless any of the donors object.

### Wants and Workers.

*Free Training.*—Is there any hospital which would give a year's free training and a certificate to a doctor's widow who has for two years earned her living as a district nurse? Excellent references.—*Alpha.*

*Home Wanted.*—Is there any retired nurse near London who could board and lodge, for a small sum, two ladies, one of whom is an invalid? —Q.

### GOD'S HAND IN ALL THINGS.

WE have been thinking lately of the goodness of God in keeping us from the rough assaults of the enemy of souls, and the temptations of the world, by drawing us aside, and by sickness or other afflictions teaching us to trust in Him alone. And who else is there we can choose for our guide? "God is a very present help in trouble;" therefore, we will not fear though the earth be shaken, and though the mountains be carried into the midst of the sea. He created us and all the world; is He not able to keep what He has made? He might have thrown us away many times when we have been disobedient; but He despises not the work of His hands, and would not have one sinner to perish. We will be contented, then, to know that we are in God's keeping; that all He does is for our good. We may wish we were in different circumstances, enjoying good health and plenty, but,

"We came not to this place by accident,
It is the very place God meant for us,"

so these quiet days we will use in the way He loves best. We will pray to Him in meekness and humility, like a little child kneeling at its mother's knee, asking for our daily bread, asking to be kept from temptation, beseeching a blessing upon our weak prayers and strength to bear whatever He sends upon us. We may not have used ourselves to look to our Heavenly Father for all things, but we will begin now, and the effort will be daily less and less. He does not ask more than we are able to do; He does not require a long string of petitions when our minds and bodies are so weak we can scarcely control our thoughts, but a few simple words for pardon, "God be merciful to me a sinner," a cry for help and strength to bear pain, these will never be in vain. He hears and answers, for He loves to see His children depending on Him. With the answer comes encouragement to persevere, for until we have tried we cannot realise the comfort which follows on an entire surrendering of our wills. To know that all things are working together for good, whether we see it or not, is bliss untold. The Holy Spirit teaches us at these times, lightens and warms us with heavenly fire, and strengthens with the fervour of Divine love.

It is the Redeemer's hand which upholds, while it seems to cripple, which strengthens while it seems to exhaust. He keeps the fainting soul in life to trust in Him.

"Then welcome each rebuff
That turns earth's smoothness rough,
Each sting that bids not sit nor stand but go,
Be our joy three parts pain!
Strive and hold cheap the strain,
Learn, nor account the pang; dare, never grudge the throe."

### A Nurse in Natal.

(*Continued from page* lii.)

As the surroundings of Miss Goodricke's second patient were very characteristic of life in Johannesburg at that time, a brief description of them may not be uninteresting.

The sick man was a bachelor in the prime of life, who, though not at all in indigent circumstances, has been obliged (owing to the crowded state of the city) to put up with a small verandah room in an hotel kept by an unmarried man, and frequented only by men. While occupying this room, he had been taken ill, and was now in a critical stage of enteric fever. On Miss Goodricke's arriving to take over the case she found him lying in a state of great exhaustion, on an old tressle bedstead, with a hard straw mattrass not in the highest state of cleanliness. There was a box beside his bed containing his clothes, and another against the wall which did duty as wash-stand, toilet table, &c. This was the only furniture the room contained; nor was there indeed space for more, a narrow passage between the bed and box table affording the only means by which the nurse could approach her patient. A small one-paned window which could not open, completed the comforts of this little den, for which the present occupant paid (including his board) the sum of £12 per month, and was considered fortunate in being able to secure even such a place as this.

After nursing for about a fortnight in this unwholesome atmosphere, Miss Goodricke was herself taken ill. The doctor, during his usual visit one morning, noticed symptoms of approaching illness, and peremptorily ordered her home. Arriving there, she had to undergo a sharp attack of fever. Fortunately for herself, however, she quickly rallied under the skilful treatment and kind attention shown to her at the institute; and so great was the demand for nurses at that time, that when she had only been a few days convalescent she was sent to take charge of another case. It would be tedious to relate the details of the busy life upon which she had now entered. Case succeeded case with such rapidity that she was often carried off by the doctors from one patient just entering convalescence to another more seriously needing care. There were at this period about one hundred doctors and twenty trained nurses in the city, and amongst all the bustling, hurrying crowd which inhabited it, none led a busier life than they. This was, perhaps, the happiest time Miss Goodricke had known since her brother's death. She was now able to throw herself with undivided attention into the work which had for her such an absorbing interest. She had the happiness of knowing herself engaged in one of the highest and noblest missions a woman can fulfil, and she had beside the satisfaction of feeling that she possessed the thorough appreciation and lively gratitude of her patients, as well as the approved and hearty co-operation of the medical profession. What I am now relating of one individual applies, of course, to all. Whether it was that the people in Johannesburg being, for the most part, newly gathered together from larger and more enterprising countries than Natal, possessed a greater share of intelligence and energy, or that the doctors themselves were more energetic in their support of the nurses I do not know, but certain it is that there was evinced the keenest appreciation of a good nurse in times of sickness. Every nurse in the city was eagerly sought after, and her services gratefully recompensed.

It seems strange that a new settlement like Johannesburg, far from all centres of civilization, and certainly possessing in some respects some of the roughest conditions of life, should yet be capable of a spirit so much more advanced and intelligent than the older and presumably more civilized country of Natal.

Yet, that such was the case, what I have still to relate will show. Miss Goodricke had not been many months in Johannesburg when the new Home for Nurses was commenced in Government Square, and rapidly brought to completion. This was built on a much more comprehensive plan than the temporary institute. It consisted of two large houses, one for the accommodation of the nurses, and one for the reception of patients who could not be efficiently nursed in their own lodgings. There was also a large separate ward for the latter purpose. This establishment was a boon to Johannesburg, and the large staff of nurses which it kept up found constant employment both in and out of doors. When she had been about two years in the city, and while she was at the very height of her busy life, a great misfortune befel Miss Goodricke. She had had a number of trying and anxious cases succeeding each other; the last and most notable of which was that of a lady who had nursed three of her own children through enteric fever, and had herself fallen a victim to the same disease after burying two of them. During the eight weeks of her illness she passed through every possible stage and complication of that terrible malady, and on her recovery was known and spoken of as the "Johannesburg Miracle." While nursing this trying case, in which she had no assistance, owing to the great amount of sickness prevalent at the time, Miss Goodricke passed many nights together without removing her clothes, and so absorbed in it did she become, that it was sometimes impossible to persuade her to leave her patient's bedside for a few hours' rest.

She felt as though every faculty of mind and body were enlisted in the struggle to withhold her patient from the jaws of Death; and it was just as convalescence had set in, and the unnatural tension was relieved that she fainted one day while kneeling beside Mrs. Helstone's bed. She was now compelled to rest, and leave her patient in other hands; and, at her own request was removed to the house in which Mrs. Thornton had a large and comfortable apartment. Then a long and trying illness succeeded, for it appeared that she had long had a neglected cold, which now in the enfeebled and over-wrought state of her nervous system asserted itself into a sharp attack of pnuemonia. Well was it for Mary that she now had her loving and faithful aunt by her side; for as the violence of disease diminished, the greatest prostration of mind and body set in. This was increased by the decision of three doctors who met in consultation over her case.

They declared that if she valued her life she must leave Johannesburg as soon as her strength permitted. She must return to Natal, and settled in one of its midland districts.

This was indeed terrible news for Mary. She must give up her beloved work in a place where she had been able to follow it with satisfaction and pleasure to herself, and the approbation of those under whom she worked.

Indecision, however, was not to be allowed her, for a constant recurrence of threatening symptoms as the season advanced, warned her to act upon the advice of her medical attendants; and so, before the winter had fairly set in, she turned her back (accompanied, of course, by Mrs. Thornton) upon the "Golden City," with many tears and heartfelt regrets. It had been to her a happy field of work and interest. She had met with the greatest kindness and encouragement from all classes, and had formed there some sincere friendships. Indeed, had Miss Goodricke been differently constituted, she might have formed still closer ties. But Mary was not like other girls; she seemed unfitted by nature for finding happiness or satisfaction in any but her own state and work, and she therefore left the Transvaal as I dare say she will remain to the end of her days—a single woman!

(*To be continued.*)

### Presentation.

Upon Miss Dewing resigning her post of Matron of the Eastern Hospital, Homerton, she has been the recipient of numerous presents from her staff. The nurses presented her with a handsome travelling bag, accompanied by an illuminated address, expressing their regret at her departure and good wishes for her future welfare. The other presents from the servants are numerous and well chosen.

### Everybody's Opinion.

[*Correspondence on all subjects is invited, but we cannot in any way be responsible for the opinions expressed by our correspondents. No communications can be entertained if the name and address of the correspondent is not given, or unless one side of the paper only be written on.*]

### HER MAJESTY'S NURSING SISTERS.

"ANOTHER OLD FOGIE" writes: Will you allow me to make a few comments on "Our Indian Letter," inserted in your paper of the 21st inst. Doubtless it was written in a kindly spirit, and much of the advice given will be of use to those for whom it is intended. It is to the two last paragraphs that I wish to draw your attention. "Plenty of silk, suede white, grey or tan gloves, riding habits, dinner and ball dresses," and even the neat uniform transformed into "silk, with red velvet belts and facings." Well may people exclaim, If these are for nurses, what is nursing coming to? It sounds almost like an invitation to those who want good times to go out and join them. Surely there are already those in our ranks who think lightly enough of their calling, and are ever striving (as it would appear by their actions) to what extent they can put it aside. The last paragraph strikes one as even worse: "Don't be frivolous, or fast, or a flirt, or disagreeable," because it not only gives the service a bad name, but your reputation will stick to you, and you will be ignored instead of being made much of by the officers and their wives, whom you meet on your voyage out. Are these ladies who are being so addressed? Are they refined, educated women? Are they among those who have undertaken the noble profession of nursing (whether as a means of gaining a livelihood or not), at least with an honest desire to be of use in the world, to assist in lessening suffering and to render all womanly assistance to the afflicted and distressed? Surely to such advice like this must sound somewhat out of place! Act like a lady because it gives the nursing in the service a bad name if you do not. If any require such advice they have certainly mistaken their calling, and possibly had a woman been head of the army nursing they would never have entered it. Act like a lady, because you wish to be made much of by officers and their wives! Is such the goal to which Her Majesty's Indian Nursing Staff aspires? No; the thing is impossible. There are as devoted nurses in Her Majesty's service, whether at home or abroad, as anywhere else, and knowing this, I could not let "Old Fogie's" letter pass unnoticed.

### LADIES IN ASYLUMS.

"TWOPENNY" writes: On looking over the last "Hospital Annual" I see in the paragraph on "Asylum Attendants," the great dearth of ladies among the nurses of the insane is deplored. May I call your attention to the fact that in the lunatic hospital, where I am an official, we have eighteen lady nurses, and instead of the others being drawn from the "domestic servant class" they are principally daughters of tradesmen or farmers, many of them being well educated. In our infirmary the nursing is careful, charts, &c., being kept, and the cases of the various invalids being daily written. There are lectures which the staff attend with enthusiasm, and all who became candidates for the last examination held by the Medico-Psychological Association, passed creditably. We have frequent entertainments got up expressly for the staff, the leave is also most liberal, each nurse getting a total of fifty-two hours monthly, besides annual leave which varies according to capacity and length of service.

[We are sincerely glad to hear on all sides of the increased refinement and training of asylum attendants. We know it is a fact that improvements in this direction are being made almost daily.—ED.]

### Appointment.

[It is requested that successful candidates will send a copy of their applications and testimonials, with date of election, to THE EDITOR, The Lodge, Porchester Square, W.]

INDIAN NURSING SERVICE.—Miss Fanny J. Harris is appointed a Sister in the Indian Nursing Service, and sails for India by troopship on December 12th. Miss Harris trained at University College Hospital; her eldest sister was one of the first lot of nurses who went out, under Miss Loch, from St. Bartholomew's Hospital in 1888, and is now promoted to be Acting Superintendent at Peshawur.

### Notes and Queries.

TO CORRESPONDENTS.—1. Questions or answers may be written on post-cards. 2. Advertisements in disguise are inadmissible. 3. In answering a query please quote the number. 4. A private answer can only be sent in urgent cases, and then a stamped addressed envelope must be enclosed. 5. Every communication must be accompanied by the writer's full name and address, not necessarily for publication. 6. Correspondents are requested to help their fellow nurses by answering such queries as they can.

**Query.**

(15) Can any readers give the address of home or hospital where a poor man suffering from long standing bronchitis could be received for a few months free of charge? A most deserving case.—*Mother Carey.*

**Answers.**

*Agnes.*—Special probotioners at St. Bartholomew's pay 13 guineas a quarter. They must be between 22 and 40 years of age. They are lodged in King's Square.

*J. H.*—A drawing of an army sister's cap will appear in these pages shortly. We do not know where you can get the pattern.

*Nurse.*—No hospital will receive as a probationer a girl of 16. Twenty is the earliest age, and then only in children's hospitals.

*Enquirer.*—There is no "active service" in connection with the St. John's Ambulance Association. If you wish to nurse the wounded in time of war, you must train three years in a hospital, and then join the ranks of the Army Sisters. Heroines are made by a long and painful process in these modern days.

*Travelling.*—Our correspondent at Leeds had better advertise in the *Morning Post*, the *Lancet*, and in our columns, if she wants an engagement to travel as nurse.

*M. J. K.*—We are unable to use your lines.

*Red Cross.*—We fancy you wish to know how to become an Army Sister. Train three years, and then apply to the Director-General, Army Medical Department, Victoria Street, S.W. See answer to *Enquirer*. There is a Red Cross Institution in Dublin, but it has nothing to do with nursing in time of war.

*Corrections.*—The appointment lately chronicled to Gainsboro' Hospital should have read Guisborough Hospital. Nurse Cocks and Nurse Carwood were lately wrongly described as Cox and Garwood.

*Christmas Competitions.*—Parcels received from "One of the First Thousand," A. Graham, Nurse Meredith, Nurse Wood.

*Consumption of the Bowels* is tuberculosis of the bowels. The activity of the tubercle bacillus is probably the exciting cause. The remote cause is generally a congenital tendency, or some adverse circumstance giving rise to general exhaustion. The only rule for the nurse in such a case is to follow the instructions of the medical man in attendance.

*Friar's Balsam* is the well-known compound tincture of Benzoin. Its other names are "Wound Balsam," "Commander's Balsam," and "Jesuit's Drops."

### Amusements and Relaxation.

**SPECIAL NOTICE TO CORRESPONDENTS.**

**Fourth Quarterly Word Competition commenced October 3rd, ends December 26th, 1891.**

Competitors can enter for *all* quarterly competitions, but no competitor can take more than *one first prize* or *two* prizes of any kind during the year.

Proper names, abbreviations, foreign words, words of less than four letters, and repetitions are barred; plurals, and past and present participles of verbs, are allowed. Nuttall's *Standard* dictionary only to be used.

N.B.—Each paper must be signed by the author with his or her real name and address. A *nom de plume* may be added if the writer does not desire to be referred to by us by his real name. In the case of all prize-winners, however, the real name and address will be published.

The word for dissection for this, the TENTH week of the quarter, being

"PRINCESS."

| Names. | Nov. 28th. | Totals. | Names. | Nov. 28th. | Totals. |
|---|---|---|---|---|---|
| Lightowlers | 68 | 415 | Jenny Wren | 64 | 327 |
| Bonne | 74 | 433 | Darlington | 70 | 410 |
| Morico | 75 | 475 | Nurse G. P. | — | 99 |
| Robes | — | 143 | Hetty | 72 | 327 |
| Dulcamara | 68 | 422 | Janet | 47 | 346 |
| Psyche | — | 7 | Jackanapes | 56 | 326 |
| Agamemnon | 63 | 445 | Ex-Nurse | — | 99 |
| Nurse J. S. | 57 | 397 | | | |

All letters referring to this page which do not arrive at 140, Strand, London, W.C., by the *first post on Thursdays*, and are not addressed PRIZE EDITOR, will in future be disqualified and disregarded.

### Off Duty.

### "Dr. Sutters."

*(Concluded from page liv.)*

Dr. Sutherst and Mabel went up the lane, and through the little white gate to the surgery.

"Now, I must warn you that it will hurt," said Dr. Sutters. "Will you have a glass of wine?"

"No, thank you. I'll trust you to be as quick as possible." Then she bared a shapely white forearm, set her teeth, and closed her eyes. Not so much as an "Oh" escaped her when the burning, biting stuff touched the naked nerves, though her face was bloodless and her lips twitched.

"Bravely borne!" said the doctor. "Now, don't be anxious about the bite; you'll be alright."

"Do you think the dog is mad?"

"No, but he's a ferocious beast. I shall certainly have him destroyed."

"Was it necessary to cauterise the wound?" she asked.

"It is always safest. Let me bind your arm. I'll come and see you in a day or two."

"Oh, no, please! you mustn't do that. I wouldn't let papa know I have been bitten—not on any account. He is dreadfully nervous about hydrophobia. His brother was bitten once when a child, and my father, who was with him, seems to have been much alarmed. I would not let him know for anything."

"Well, then, you must come here."

"Thank you, I would much prefer it, if you have no objection."

"Oh, none whatever."

"Thank you very much. You will promise, please, not to tell anyone."

"Certainly."

"Thank you so much."

With a friendly, grateful smile, she went out of the surgery, while the doctor held the door open and bowed. From the blind he watched her walk down the garden path, while he muttered: "A plucky girl, 'pon my word! How pretty, too! I could hardly see her in the twilight the other evening. Ah; my young lady, I could have made you dance just now if I had chosen. I could have had a pretty revenge for that slander on my nose, and that flippancy about my professional skill."

Two days after, Mabel came to show her arm.

"Going on well," said Dr. Suthers, "Has the secret leaked out?"

"No; no one knows—not even my sisters. I hope you don't mind being a party to this conspiracy of silence?"

"Oh, we can tell Mr. Norton one day. I am glad I came up in time the other day."

"Yes; how very fortunate it was. I'm sure the wretched dog would never have let go. I am very grateful to you, Dr. Sutherst."

His grey eyes twinkled for a moment through his glasses. "'Old Sutters' is getting on," he laughed. "He has another case. What do you think of his treatment?"

Flushing rosy, she looked down at her glove button, and said—

"Really, Dr. Sutherst, you mean to punish me for my silly sketch of you. I—I know it was spiteful, but——"

"You couldn't help it, I suppose?' he interrupted. "You took a dislike to me the first time you saw me. Yes, yes; I *know* you did."

"Oh, Dr. Sutherst, why do you say that?"

"I read it on your face, and t was confirmed when I came to the Vicarage. Why did you exaggerate my nose so cruelly? I'll own it is attenuated, but it's not blue. Also, I will not deny that my hair is sandy, but it isn't a vivid red. There was aversion in every line of that drawing."

"Why should I hate you when I didn't know you?"

"I don't know, unless it was my appearance," he chuckled.

"Oh! really, Dr. Sutherst, I had no intention of being unkind. Do, *do* forgive me?" Her eyes met his, and humour and contrition were oddly mingled in their expression.

"I have nothing to forgive," he answered. "I have learned a lesson. I was inquisitive. They say 'listeners hear no good of themselves,' and in the same sense the axiom applies to peepers at drawings. I thought it very probable that you had caricatured me. My face lends itself to that sort of portrait. I looked, and—and I was punished. But, really, you have a genius for that work."

"You flatter me. Those sketches are hasty, and only intended for my own delectation."

"Well, you could scarcely expect your subjects to take a great delight in them, when you lay such stress upon their facial blemishes. Will you give me that sketch?"

"I tore it up yesterday."

"Tore it up! I'm sorry to hear that. Won't you do another?"

"I am afraid I can't," she said, with a smile. "I couldn't put any spite into the execution."

Then the conversation merged into the topic of beetles, and Mabel begged to see the famous collection. This request quite won the doctor's heart. He opened drawer after drawer, and explained the Latin names. A full hour passed, and their heads were very near together as they bent over the cabinet.

From that day Dr. Sutters began to practise the social virtues by calling regularly at the Vicarage, and cultivating a friendship with Mr. Norton. He chalked the tennis lawn for the girls, and even consented to wield a racket; in short, he blossomed into a ladies' man in a remarkably short space of time. One day he was seized with the idea that Mabel's talent in water-colour painting might be turned to practical account. She should illustrate the great work on the Coleoptera. Mabel was immensely pleased with the suggestion.

"How lovely," she exclaimed. "And may I come up to the Firs to copy the beetles?"

"Of course they must be drawn from the originals," he said. "But I make one stipulation—you must not caricature them."

"Will you never forgive me?" she said humbly.

It was a warm July night, and hay and honeysuckle scented the air, as they strolled at the end of the Vicarage garden. Dr. Sutters's face glowed joyfully in the moonlight, and the soft felt hat was pushed back from "the massive brow."

"Accede one condition, Mabel, and I'll never speak about the caricature again," he said.

"What is it?" she asked.

"That you marry 'old Sutters,' as a reparation for the cruel wrong you have done to his facial organs—the nose in particular. I love you dearly, Mabel. I am not so very ancient, either; though I look fifty-six, I'm only forty-eight. I think I can make you happy, and I'm sure we understand each other by this time I meant to live on in selfish bachelorship, until recently. But you have awakened my torpid emotions. Yes, I am in love. Come, do you think I shall make a passable husband? Don't leave 'old Sutters' alone with his beetles."

"I know you will make more than a 'passable husband,'" she said. "No, I won't leave you to the beetles; they are gradually demoralising you. I hope I shall be able to make you happy, Dr. Sutherst. I am afraid I'm not sedate enough."

"Tush! you are just the element that is needed in my drab, lonely existence," said the doctor, resting his hands upon her shoulders, and looking tenderly at her eyes.

## Figures and Tables

**Figure f1:**